# Semaglutide in Diabetic Kidney Disease: Integrating Clinical Evidence with Mechanistic Insights

**DOI:** 10.3390/healthcare13222922

**Published:** 2025-11-14

**Authors:** Faten F. Bin Dayel

**Affiliations:** Department of Pharmacology, College of Pharmacy, Prince Sattam Bin Abdulaziz University, Al-Kharj 11942, Saudi Arabia; f.bindayel@psau.edu.sa

**Keywords:** semaglutide, diabetic kidney disease, GLP-1 receptor agonists, renoprotection, type 2 diabetes mellitus, chronic kidney disease

## Abstract

Semaglutide, a glucagon-like peptide-1 receptor agonist (GLP-1RA), has demonstrated substantial efficacy in managing type 2 diabetes mellitus (T2DM). It provides glycemic control, promotes weight loss, and offers cardiovascular protection. Evidence also supports its role in diabetic kidney disease (DKD), a leading global cause of end-stage renal disease. DKD arises from a multifactorial interaction involving hyperglycemia, hypertension, and inflammation, which leads to cumulative nephron loss. Beyond glycemic control, semaglutide’s mechanisms of action target metabolic and hemodynamic pathways that contribute to renal damage. This review evaluates the preclinical and clinical evidence of semaglutide’s role in preventing DKD, focusing on its renal effects and the mechanistic basis for renoprotection. We also position semaglutide within the broader DKD therapeutic landscape by reviewing clinical trial findings, translational studies, real-world evidence, and its effectiveness compared to other drug classes. The expanded actions of semaglutide make it a promising agent in patients with T2DM and DKD and encourage further mechanistic research and long-term evaluation.

## 1. Introduction

Diabetic kidney disease (DKD) is a microvascular complication of diabetes mellitus. It is currently the leading cause of chronic kidney disease (CKD) and end-stage renal disease (ESRD) globally. As the number of type 2 diabetes mellitus (T2DM) cases is rising, the prevalence of DKD is also increasing. DKD is primarily linked to morbidity, mortality, and rising healthcare costs [1,2], and has been observed to develop in about 20–50% of individuals with T2DM. The features of DKD are persistent albuminuria, a reduced glomerular filtration rate (GFR), and an increased cardiovascular risk [3,4]. Despite advances in glycemic control, renin–angiotensin system blockade, and blood pressure and lipid management, the residual risks of renal and cardiovascular issues remain high [5,6].

Pharmacological treatment for T2DM has changed significantly in the past decade. New drugs now provide additional glucose-lowering benefits. Among these, glucagon-like peptide-1 receptor agonists (GLP-1RAs) are highly prioritized as they provide cardiovascular protection, weight reduction, and anti-inflammatory effects [7,8,9]. Once-weekly administration of GLP-1RA semaglutide more effectively reduces glycated hemoglobin (HbA1c) levels, body weight, and cardiovascular events than traditional agents [9,10]. Notably, new evidence suggests that it may provide renoprotective effects [11]. Semaglutide stands out among GLP-1 receptor agonists for its advanced pharmacologic profile and consistent renal benefits. Its molecular design exhibits enhanced albumin binding and structural modifications that prolong its plasma half-life, enabling once-weekly subcutaneous dosing, while the oral formulation is taken once daily [12,13]. This extended exposure sustains receptor activation and improves glycemic and extra-glycemic efficacy compared with other agents. For example, liraglutide requires daily injection, while dulaglutide, though administered weekly, leads to smaller reductions in HbA1c and body weight [13,14]. Data from the SUSTAIN-6 trial demonstrate that semaglutide exerts renoprotective effects, predominantly through the attenuation of new-onset macroalbuminuria [15]. By contrast, the renal outcomes observed after liraglutide (LEADER trial) and dulaglutide (REWIND trial) administration were favorable but of comparatively lower magnitude [16,17]. Collectively, these findings suggest that semaglutide’s molecular modifications translate into clinically meaningful renal protection beyond glycemic control, distinguishing it within the GLP-1RA class. This review aimed to assess the literature on semaglutide in DKD. We summarize its pharmacological properties, actions, and recent clinical and mechanistic evidence, evaluating its promise as a disease-modifying therapy for diabetic nephropathy.

## 2. Semaglutide

Semaglutide is a weekly administered glucagon-like peptide-1 receptor agonist (GLP-1RA) used in the treatment of type 2 diabetes mellitus (T2DM) as well as obesity. Semaglutide is 94% homologous with endogenous GLP-1 but contains the necessary modifications to offer an enhanced stability and half-life [18]. Particularly, substitution at position 8 in alanine with α-aminoisobutyric acid confers resistance to degradation by dipeptidyl peptidase-4 (DPP-4), and acylation with C18 fatty acid enables albumin binding and systemic exposure over prolonged periods [13,19]. These structural changes allow for once-weekly dosing, a regimen that has been demonstrated to greatly increase drug persistence and adherence, thereby reducing patient treatment burden and potentially maximizing the therapeutic outcomes in real-world clinical practice [20]. The pharmacological effects of semaglutide are mediated by GLP-1 receptor activation on pancreatic β-cells, resulting in increased glucose-dependent insulin secretion and suppression of glucagon release. Semaglutide decreases gastric emptying and enhances satiety via central hypothalamic mechanisms, leading to significant weight loss [21,22]. Importantly, these effects are not accompanied by a high risk of hypoglycemia [23]. In SUSTAIN 5 and SUSTAIN 7 clinical trials, semaglutide consistently decreased HbA1c by 1.1% to 1.8% and body weight by 4–6 kg in diverse populations [14,24]. In SUSTAIN-6, a historic cardiovascular outcomes trial, semaglutide reduced major adverse cardiovascular events (MACEs) by 26% relative to a placebo [9]. Semaglutide had kidney-protective effects in patients with T2DM, which were more pronounced in those with baseline chronic kidney disease [11]. Semaglutide has a similar safety profile to other members of the GLP-1RA class, the most common adverse events being gastrointestinal effects such as nausea, vomiting, and diarrhea, particularly on dose escalation. These findings are mirrored in randomized controlled trials (RCTs) and further corroborated by post-marketing pharmacovigilance experience [25,26]. Available data from clinical trials and post-marketing experience have not demonstrated a consistent, causal association between exposure to semaglutide and the development of acute pancreatitis or medullary thyroid carcinoma (MTC) in humans. However, in rodents, it causes thyroid C-cell tumors; therefore, this contraindicates its use in patients with a personal or family history of MTC or Multiple Endocrine Neoplasia syndrome type 2 (MEN2) [27,28]. When used alone or in combination with metformin, semaglutide has an inherently low hypoglycemia risk, but this increases when used with insulin or with insulin secretagogues such as sulfonylureas [23]. Diabetic retinopathy complications are a rare but serious problem, as observed in SUSTAIN-6, though subsequent analysis suggests that this is more a consequence of too-rapid glycemic correction than a drug effect per se [29,30].

## 3. Diabetic Kidney Disease

Diabetic kidney disease (DKD), or diabetic nephropathy, is a chronic disease of both type 1 and type 2 diabetes mellitus, where the latter is the most significant contributor due to its global prevalence. DKD is responsible for about 30–50% of all cases of end-stage renal disease (ESRD) in developed nations. It is also associated with very high cardiovascular morbidity and mortality [2,31]. Despite advancements in managing diabetes, DKD is continuing to increase in prevalence, particularly among older adults and obese individuals [32]. Pathologically, DKD is characterized by initial glomerular hyperfiltration, followed by microalbuminuria, macroalbuminuria, and finally a reduced estimated glomerular filtration rate (eGFR). Histologically, it is characterized by mesangial matrix expansion, glomerular basement membrane thickening, loss of podocytes, arteriolar hyalinosis, and interstitial fibrosis [33,34]. These alterations are induced by chronic hyperglycemia, oxidative stress, activation of the renin–angiotensin–aldosterone system (RAAS), and pro-inflammatory and profibrotic pathway upregulation, especially through transforming growth factor-β (TGF-β) and nuclear factor-kappa B (NF-κB). Hyperinsulinemia, insulin resistance, dyslipidemia, and sodium retention also contribute [35,36]. Clinically, a diagnosis of DKD is most commonly established based on persistent albuminuria (albumin-to-creatinine ratio > 30 mg/g), a declining eGFR (<60 mL/min/1.73 m^2^), or both, excluding other renal disease etiologies [37]. Nevertheless, 56.6% of T2DM patients with DKD may have a decreased eGFR without albuminuria, an indication of the heterogeneity of disease phenotypes [38].

## 4. Semaglutide’s Effect on Diabetic Kidney Disease

### 4.1. Mechanistic Basis for Renoprotection

Experimental and translational data suggest that GLP-1 receptor agonists modulate multiple biological processes implicated in the pathogenesis of DKD. These include oxidative stress, inflammation, fibrosis, and endothelial dysfunction. Chronic hyperglycemia is the main determinant of the metabolic and molecular disturbances that form the basis of diabetic nephropathy. It leads to overgeneration of reactive oxygen species (ROS), and consequently oxidative stress, producing an environment that damages glomerular and tubular cell function [39]. This oxidative environment also interacts with inflammatory pathways, creating a feed-forward loop that exacerbates renal injury. In combination with this redox stress, inflammatory signaling is triggered. Clinical and experimental evidence has particularly underlined the role of pro-inflammatory cytokines, including tumor necrosis factor-α (TNF-α), interleukin-1 (IL-1), interleukin-6 (IL-6), and interleukin-18 (IL-18), which exacerbate structural and functional renal injury [40]. Therefore, oxidative stress and inflammation appear to be concomitant mechanisms, where metabolic stress preconditions renal tissue for damage and cytokine-mediated cascades facilitate disease progression. In a preclinical model of renal damage related to obesity, administration of semaglutide significantly reduced TNF-α, IL-6, and IL-1β levels, with increased activity of antioxidant enzymes such as superoxide dismutase (SOD) and reduced renal malondialdehyde (MDA) [41]. This suggests that semaglutide could have a protective effect against oxidative stress and inflammation in DKD. Building on these oxidative and inflammatory mechanisms, recent research has also explored the cellular and molecular pathways through which semaglutide may exert renoprotective effects. Recent research has highlighted mechanisms in which semaglutide can exhibit its renoprotective effects in DKD. One line of evidence implicates β-Klotho (KLB) as a key mediator. This involves the cyclic adenosine monophosphate (cAMP)/protein kinase A (PKA)/phosphorylation and cAMP-response element-binding protein (CREB) signaling pathways. Semaglutide increases KLB expression and activates AMPK and thus reprograms ferroptosis-related metabolic pathways such as iron regulation, fatty acid synthesis, and antioxidant response to lipid peroxidation. The result is suppression of ferroptosis with secondary reductions in renal inflammation and fibrosis [42]. Another study demonstrated that in a model of hypertensive-accelerated DKD in uninephrectomized db/db mice with renin overexpression, treatment with semaglutide significantly improved kidney injury, including glomerulosclerosis, with a simultaneous significant improvement in hyperglycemia, hypertension, and albuminuria. It also improved the podocyte filtration slit density and reduced the levels of urine and renal kidney injury molecule-1 (KIM-1), which is a marker for kidney injury, as well as suppressing inflammatory and fibrogenic gene expression [43]. Findings support that semaglutide could have the potential to manage DKD with its actions of glycemic control and direct cellular protection, modulation of ferroptosis, and improvement of hemodynamic and inflammatory stressors.

Extending from these intracellular and metabolic effects, semaglutide may also influence intercellular communication pathways that contribute to DKD progression. Moreover, recent evidence indicates that endothelial-derived extracellular vesicles (EVs) contribute to macrophage activation and upregulation of pro-inflammatory cytokines in diabetic tissues, implicating EV-mediated inflammatory signaling in the propagation of chronic inflammation. This identifies EVs as critical intermediaries linking endothelial dysfunction with immune activation, representing an emerging mechanistic insight in diabetes and its complications [44].

**Figure 1:**
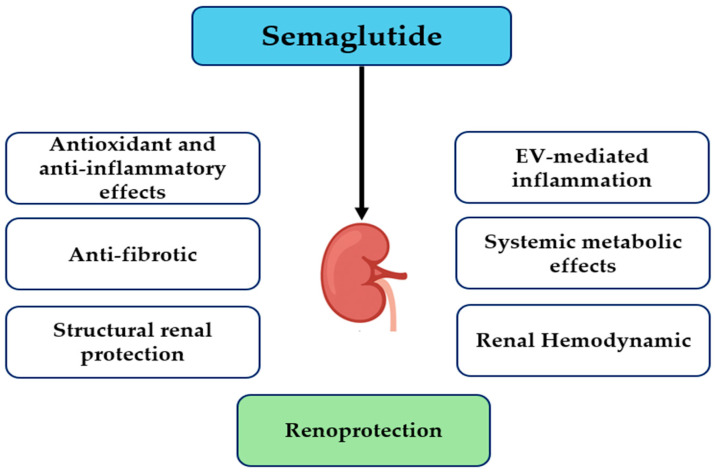
Proposed mechanisms of semaglutide-mediated renoprotection. Abbreviation: EV, endothelial-derived extracellular vesicle.

The results of McLean et al. show that semaglutide decreases atherosclerosis in mice, regardless of GLP-1 receptor expression on Tie2+ endothelial cells and hematopoietic cells, suggesting that the vascular protective effects of semaglutide are to mediated a great extent by positive systemic metabolic effects, e.g., reductions in glucose, lipid toxicity, and inflammation, but not via endothelial GLP-1R signaling. However, the same study identified Tie2+ GLP-1R signaling as important for anti-inflammatory and antifibrotic action in the liver, which correlates with tissue-specific variances [45]. This is consistent with the hypothesis that semaglutide could indirectly improve endothelial dysfunction and albuminuria in DKD through systemic metabolic and anti-inflammatory actions, but not through direct endothelial receptor interactions. Finally, linking these systemic effects to renal hemodynamics further illustrates semaglutide’s multifaceted renoprotective profile. A study conducted by Farah et al. showed that GLP-1 receptor signaling plays a crucial role in the regulation of renal sodium handling, primarily by phosphorylating and inhibiting the proximal tubule Na^+^/H^+^ exchanger NHE3 via cAMP/PKA-dependent pathways, thereby increasing natriuresis and diuresis [46]. While Farah et al. studied endogenous GLP-1 rather than semaglutide specifically, these mechanisms provide a probable explanation for how GLP-1RA-like semaglutide may influence renal hemodynamics, reduce intraglomerular pressure, and provide an additive renoprotective effect, particularly in the hyperfiltration setting, a characteristic of DKD. These mechanisms are summarized schematically in Figure 1, which illustrates the major cellular and molecular pathways implicated in DKD that are targeted by semaglutide. A concise mapping of these pathways, linking preclinical and clinical evidence, is provided in Table 1.

### 4.2. Clinical Trial Evidence

Data for the renoprotective actions of semaglutide in humans have been extracted from several randomized clinical trials and post hoc analyses (Table 2). Although not originally designed to assess the renal effects of semaglutide as part of the primary endpoints, except for FLOW trial, the prespecified analyses have yielded important information. In SUSTAIN-6, a 3297-patient randomized double-blind trial of type 2 diabetics at high cardiovascular risk, semaglutide reduced the risk of new or worsening nephropathy by 36% (HR 0.64, 95% CI 0.46–0.88). This effect was explained in large part by a 46% reduction in new-onset persistent macroalbuminuria (HR 0.54, 95% CI 0.37–0.77), but there were also nonsignificant trends towards a lower decline in eGFR and fewer cases of serum creatinine doublings [9]. A pooled analysis of the SUSTAIN 1–7 programs confirmed these findings, showing attenuated eGFR declines across diverse patient populations without new safety concerns [47].

Oral semaglutide has also demonstrated renal safety and potential renoprotection. In PIONEER-5, involving patients with T2DM and moderate CKD, oral semaglutide was effective, well tolerated, and associated with stabilization of kidney function, including mild reductions in albuminuria [48]. In PIONEER-6, designed primarily as a cardiovascular outcomes trial, renal endpoints were exploratory. Nonetheless, a post hoc analysis revealed 36% fewer nephropathy events (HR 0.64, 95% CI 0.46–0.88) and a 46% lower risk of new-onset macroalbuminuria (HR 0.54, 95% CI 0.37–0.77) compared with a placebo, paralleling the pattern seen with injectable semaglutide [15]. Collectively, these findings support a class-wide renoprotective effect of GLP-1 receptor agonists rather than formulation-specific effects.

A systematic review and meta-analysis of seven GLP-1RA cardiovascular outcome trials by Sattar et al., 2021 [49] showed a consistent 21% reduction in composite kidney outcomes with GLP-1RA use, largely driven by decreases in macroalbuminuria. Semaglutide, however, appeared to show a quantitatively greater effect than other agents, including liraglutide and dulaglutide, although the mechanism for this difference has not been firmly established. Overall, while evidence from clinical trials regarding the effects of semaglutide in the kidneys is compelling, it is not yet exhaustive. The FLOW trial should provide definitive clarity on semaglutide’s role as a disease-modifying therapy in DKD.

Larger, long-term renal outcome studies were previously required to confirm these preliminary observations. To surmount these limitations, the FLOW trial (NCT03819153), the first renal-dedicated outcomes trial for a GLP-1 receptor agonist, has now reported its primary results [50,51]. This global, randomized, double-blind, placebo-controlled trial evaluated once-weekly subcutaneous semaglutide (1.0 mg) versus placebo in 3533 patients with T2DM and CKD (eGFR 25–75 mL/min/1.73 m^2^ and UACR 100–5000 mg/g). A 24% reduction in the primary composite outcome of sustained ≥ 50% reduction in eGFR, ESKD, or death from renal or cardiovascular causes (HR 0.76; 95% CI 0.66–0.88) was observed during a median follow-up of 3.4 years [51]. The risk of kidney failure alone was reduced by 24%, and the risk of major adverse cardiovascular events by 18%, with consistent safety findings compared with the placebo. As the first stand-alone renal outcomes trial for a GLP-1 receptor agonist with hard renal endpoints, FLOW establishes robust evidence that semaglutide slows kidney disease progression in patients with T2DM and CKD, beyond its glucose-lowering effect. These findings align with previous meta-analyses and pooled analyses supporting the renoprotective actions of GLP-1 receptor agonists.

**Table 2:** Summary of clinical trials and post hoc analyses evaluating renal outcomes with semaglutide in patients with T2DM and DKA.

Trial	Study Design	Population	Intervention	Duration	Renal Baseline Characteristics	Primary Renal Outcome	Key Findings	**Reference**
SUSTAIN-6	Randomized, double-blind, placebo-controlled	T2DM with high CV risk	Semaglutide 0.5–1.0 mg weekly vs. placebo	Median 2.1 years	Mean eGFR: 75.6 mL/min/1.73 m^2^; UACR 34 mg/g	Combination of new/worsening nephropathy (≥30% eGFR decline, ESKD, or death from renal causes)	36% reduction in nephropathy events (HR 0.64, 95% CI 0.46–0.88); 46% lower risk of macroalbuminuria (HR 0.54, 95% CI 0.37–0.77); slower eGFR decline vs. placebo	[9]
SUSTAIN 1–7 pooled analysis	A pooled post hoc analysis of the SUSTAIN 1–7 randomized controlled trials	T2DM patients	Semaglutide vs. placebo or active comparators, depending on the specific SUSTAIN trial	Varies by trial	Varies by trial	Changes in eGFR, reductions in urinary albumin-to-creatinine ratio (UACR), and the occurrence of kidney-related adverse events	Initial eGFR dip followed by stabilization, resulting in a net annual slope improvement of ≈0.59 mL/min/1.73 m^2^ versus comparators; significant reductions in UACR; no new renal safety signals observed	[47]
PIONEER-5	Randomized, double-blind, placebo-controlled phase 3a	T2DM with moderate renal impairment (eGFR 30–59 mL/min/1.73 m^2^)	Oral semaglutide (14 mg daily) vs. placebo	A 26-week randomized treatment period and a follow-up period of 5 weeks	eGFR 30–59 mL/min/1.73 m^2^ (moderate CKD)	Exploratory renal safety and albuminuria change (not dedicated renal composite)	eGFR remained generally stable in both groups (no slope provided); UACR decreased numerically in the semaglutide group (magnitude not reported, no formal statistical test); no new renal safety issues were identified	[48]
PIONEER-6	Randomized, double-blind, placebo-controlled CVOT	T2DM with high CV risk (eGFR ≥ 30 mL/min/1.73 m^2^)	Oral semaglutide (14 mg daily) vs. placebo	Median follow-up = 1.3 years	Mean baseline eGFR = 74 mL/min/1.73 m^2^ (estimated from pooled data)	Exploratory renal safety outcomes were assessed, but there was no prespecified primary renal endpoint	Exploratory renal outcomes: HRs for persistent eGFR decline <1.0 overall (NS); in patients with baseline eGFR 30–<60, semaglutide significantly reduced the risk of 30% eGFR decline (*p* = 0.03); no major renal safety concerns; no statistically different interactions between treatment and CKD subgroup were observed	[15]
Post hoc analysis of SUSTAIN-6 and PIONEER-6	Pooled post hoc analysis of two cardiovascular outcome trials (SUSTAIN-6 and PIONEER-6)	Patients with T2DM at high cardiovascular risk (n = 6480; semaglutide = 3239; placebo = 3241)	Semaglutide (s.c. and oral) vs. placebo	Median follow-up = 2.1 years (SUSTAIN-6) + 1.3 years (PIONEER-6)	eGFR ≥ 30 mL/min/1.73 m^2^; subgroup with eGFR 30–<60 mL/min/1.73 m^2^ analyzed separately; baseline UACR available for SUSTAIN-6 only	Annual eGFR slope; time to persistent eGFR decline (≥30%, ≥40%, ≥50%, ≥57%)	Annual eGFR decline was slower by 0.59 mL/min/1.73 m^2^/year (95% CI 0.29–0.89) with semaglutide vs. placebo. In patients with baseline eGFR 30–<60, the difference was 1.06 mL/min/1.73 m^2^/year (95% CI 0.45–1.67). HRs for time to persistent eGFR decline were consistently <1.0, favoring semaglutide, though not all reached statistical significance	[52]
Meta-analysis of GLP-1 receptor agonists	Systematic review and meta-analysis of 8 major cardiovascular outcome trials (n = 60,080)	Patients with T2DM; broad cardiovascular risk spectrum	GLP-1 RAs (including liraglutide, semaglutide, dulaglutide, albiglutide, exenatide, lixisenatide) vs. placebo	Median trial duration 1.3–5.4 years	Baseline mean eGFR ~78 mL/min/1.73 m^2^; ~23% with eGFR < 60 mL/min/1.73 m^2^	Combined kidney outcomes (new-onset macroalbuminuria, sustained ≥30–40% decline in eGFR, kidney replacement therapy, or renal death)	GLP-1 receptor agonists reduced the combined kidney outcome by 21% (HR 0.79; 95% CI 0.73–0.87; *p* < 0.0001), driven largely by a 26% lower risk of new-onset macroalbuminuria (HR 0.74; 95% CI 0.68–0.81). Risk reduction for hard renal endpoints (≥40% eGFR decline, doubling of serum creatinine, end-stage kidney disease, or renal death) was nominal (HR 0.86; 95% CI 0.72–1.02). Effects were consistent across baseline eGFR subgroups	[49]
FLOW	Randomized, double-blind, placebo-controlled, event-driven kidney-outcomes trial	3533 adults with T2DM and CKD (eGFR ~47 mL/min/1.73 m^2^; median UACR ~568 mg/g)	Semaglutide 1.0 mg weekly vs. placebo (plus standard of care)	Median ~3.4 years	Mean baseline eGFR = 47 mL/min/1.73 m^2^; median UACR = 568 mg/g	Composite of kidney failure, sustained ≥50% eGFR decline, kidney/cardiovascular death	24% reduction in primary composite kidney outcome (HR 0.76; 95% CI 0.66–0.88). Annual eGFR decline slowed by ~1.16 mL/min/1.73 m^2^/yr in the semaglutide group	[50,51]

### 4.3. Translational Studies and Real-World Evidence

Although randomized controlled trials (RCTs) are still the gold standard to assess drug efficacy, they are usually limited by narrow inclusion criteria, limited follow-up times, and a lack of external validity. Translational studies and real-world evidence (RWE) can potentially close these gaps through an assessment of therapy performance in more general populations as well as through the creation of mechanisms in human tissue. For semaglutide, aggregate real-world and translational evidence has started to substantiate its renal protective role.

In actual clinical practice, semaglutide treatment of patients with T2DM and CKD has been linked to reduced progression of kidney deterioration and statistically significant decreases in albuminuria. In a Spanish multicenter cohort of n = 156 patients, a substantial amelioration in the annual slope of eGFR from −3.29 to −0.79 mL/min/1.73 m^2^/year was documented, despite a follow-up duration of at least two years and the sample size of the study [53]. Another observational trial in 122 CKD patients demonstrated a mean 51% decrease in the UACR in macroalbuminuria subjects with a sustained eGFR over a period of 12 months.

Similarly, a multicenter real-life observational study of T2DM 2 and CKD (mostly stage 2 and 3) patients treated with subcutaneous semaglutide revealed that albuminuria was significantly reduced, especially among those with macroalbuminuria at baseline, and the eGFR was unchanged over 12 months of follow-up [54]. However, the majority of mechanistic and clinical world evidence comes from small, unblinded, or observational trials that did not have solid endpoints like ESKD or doubling of serum creatinine. However, they do offer considerable biologic plausibility and demonstrate that semaglutide’s kidney-protective effects are not a consequence of enhanced glycemia or weight loss. The combination of RCT data, observational data, and mechanistic research at the tissue level supports the hypothesis that semaglutide has multifactorial renal protective action on DKD. Table 3 summarizes the key distinctions between randomized controlled trials and real-world observational studies in semaglutide research.

### 4.4. Comparative Effectiveness with Other Drug Classes

The conventional therapy for DKD has been centered on intense glycemic control; reducing blood pressure, particularly with RAAS inhibitors; reducing lipids; and lifestyle modifications. All these treatments, while useful in the sense of retarding progression, are not yet able to eliminate the risk of ESRD or cardiovascular events. Large trials have substantiated this: tight glucose control has been observed to lead to a small reduction in nephropathy in the ADVANCE trial, but has a low impact on macrovascular events [55], and there was no clear renal benefit of strict glucose reduction in the ACCORD trial and it was indeed related to increased mortality in some groups [56].

Recent therapeutic advances have significantly changed the landscape of treatment. Sodium-glucose cotransporter-2 (SGLT2) inhibitors canagliflozin and dapagliflozin showed robust renoprotective effects in large cardiovascular and renal outcomes trials [57,58]. Similarly, nonsteroidal mineralocorticoid receptor antagonist finerenone led to decreases in the risk of CKD progression and cardiovascular events [59]. However, significant residual risks still exist, e.g., finerenone-induced hyperkalemia, emphasizing the need for adjunctive agents with complementary mechanisms of action. GLP-1 receptor agonists are also promising drugs in the context of lowering the rates of DKD development and progression by virtue of their anti-inflammatory, antifibrotic, and natriuretic properties, in addition to their established metabolic and cardiovascular benefits. Of these, liraglutide has shown early evidence of renal benefits in outcome trials [16], and mechanistic and translational studies also support the kidney benefits of the GLP-1RA class [60,61]. Semaglutide is increasingly mentioned as having potential renal effects. While the evidence is less extensive than for liraglutide, injectable and oral formulations have yielded promising findings. Semaglutide should be considered in the context of other renoprotective medications, both to put its efficacy into perspective and to assess any additive or complementary value it may have.

There are two other drug classes already well known for their effects of slowing the progression of DKD: SGLT2 inhibitors and MRAs. SGLT2 inhibitors such as canagliflozin, dapagliflozin, and empagliflozin have demonstrated robust and consistent renal benefits in studies like CREDENCE, DAPA-CKD, and EMPA-KIDNEY [57,58,62]. These drugs reduce glomerular hyperfiltration via afferent arteriolar vasoconstriction caused by tubuloglomerular feedback. They also lower intraglomerular pressure, albuminuria, systemic blood pressure, and renal hypoxia. In the DAPA-CKD study, dapagliflozin reduced the composite risk of a sustained ≥50% eGFR reduction, ESKD, and renal or cardiovascular death by 39% compared with the placebo, even in non-diabetic individuals [58]. For semaglutide, in contrast, the renal benefits are mostly linked to decreases in albuminuria, with less evidence (so far) for direct eGFR protection. Semaglutide also has more generalized systemic effects, including dramatic weight loss, appetite regulation, and anti-inflammatory effects. While SGLT2 inhibitors have modest effects on weight and glycemia, GLP-1RAs have shown more pronounced effects on both weight and glycemia, particularly in those with obesity or suboptimal glycemic control, highlighting their potential as a comprehensive treatment option in DKD [26]. A nonsteroidal selective MRA, finerenone, also demonstrated renal and cardiovascular advantages in patients with DKD, as seen in the FIDELIO-DKD and FIGARO-DKD studies [59,63]. Finerenone has antifibrotic and anti-inflammatory effects through mineralocorticoid receptor antagonism, leading to an average reduction of about 18% in the development of ESKD or a persistent decrease in eGFR. Nevertheless, it is associated with an elevated risk of hyperkalemia and requires more intense monitoring. Semaglutide, on the other hand, is not reportedly linked to electrolyte imbalance and is safer in that regard.

Studies have explored the co-administration of GLP-1RA and SGLT2 inhibitors in T2DM, presenting evidence of their synergistic effects for enhanced metabolic, cardiovascular, and renal benefits, as well as additive effects on weight and blood pressure without worsening their safety profiles [64,65]. This could be interrupted by SGLT2 inhibitors’ action on renal hemodynamics and natriuresis and GLP-1Ras’ action on oxidative stress and systemic inflammation. This is supported by the American Diabetes Association (ADA) and Kidney Disease Improving Global Outcomes (KDIGO) [66,67]. However, direct comparisons between semaglutide and other renoprotective medications remain limited. The FLOW trial will be the first to establish whether semaglutide can match or exceed SGLT2 inhibitors’ or MRAs’ kidney benefits. Until then, it needs to be considered as a dual therapy in DKD, rather than an alternative therapy, particularly in those with obesity, significant cardiovascular risk, or intolerance to other medications. Table 4 summarizes the comparative mechanisms of action, renal outcomes, and safety profiles of semaglutide, SGLT2 inhibitors, and nonsteroidal MRAs.

### 4.5. Future Directions

Despite emerging data, there are substantial gaps in the knowledge to be addressed prior to supporting the use of semaglutide as a foundation therapy for diabetic kidney disease. At the forefront of these is the need for conclusive evidence on long-term renal outcomes. While declines in albuminuria are promising surrogate endpoints [68], the FLOW trial (NCT03819153) has provided a definition of the impact of semaglutide on the long-term trends in eGFR, ESKD rate, and renal mortality [50].

The efficacy and tolerability in advanced CKD (eGFR < 30 mL/min/1.73 m^2^) are not well investigated. Patients with ESRD are often excluded or underrepresented, but are most likely to require efficacious renoprotective therapy. Well-considered investigations of tolerability, dosing regimens, and the impacts on volume status in this cohort are needed.

The identification and validation of response biomarkers to semaglutide—comprising urinary kidney injury molecule-1 (KIM-1), neutrophil gelatinase-associated lipocalin (NGAL), and transforming growth factor-beta (TGF-β) levels, multi-omics, and imaging—would allow for precision medicine approaches to identify patients who would gain the maximum benefit and could be used to follow response to treatment and determine semaglutide’s mechanisms. Therefore, research needs to be conducted to investigate semaglutide’s renoprotective mechanism of action in humans, specifically for its anti-inflammatory, antifibrotic, and hemodynamic action. In addition, long-term renal biopsy studies, along with new imaging technologies, would also provide information at the tissue level. Moreover, the optimal combination and timing of semaglutide with SGLT2 inhibitors and MRA need to be determined in subsequent randomized controlled trials. Whether combination therapy’s benefits are additive or synergistic, or whether it simply causes side effects, would be useful to know in practice. Lastly, real-world evidence from diverse populations, many of which are comorbid and ethnically diverse, will supplement trial data and guide generalizability. In sum, semaglutide is highly promising for DKD management, but its definitive place, optimum utilization, and maximum patient benefits have yet to be confirmed through high-quality intensive research. Table 5 summarizes the current knowledge gaps and suggested areas for future investigation.

## 5. Conclusions

DKD is a serious clinical issue that has high morbidity and mortality rates and is a large burden on healthcare systems, even with improvements in overall glycemic and blood pressure control. Semaglutide has shown promise as an agent not only for glycemic control and weight reduction, but also cardiovascular and renal protection. Preclinical evidence suggests that semaglutide has effects beyond the primary pathways of DKD pathogenesis, including effects on inflammation, oxidative stress, fibrosis, and endothelial dysfunction. In the clinic, cardiovascular outcome trials have demonstrated significant decreases in new or worsening nephropathy, driven primarily by decreased albuminuria.

Although the FLOW trial provides definitive evidence of semaglutide’s potential to retard kidney function decline and prevent ESKD, evidence in the literature indicates that it could be a valuable adjunct or alternative to current renoprotective agents such as SGLT2 inhibitors and mineralocorticoid receptor antagonists. Semaglutide’s pleiotropic effects, beneficial safety profile, and potential for combination therapy make it a candidate for managing combined diabetes and kidney disease. However, critical gaps in knowledge remain, such as its effectiveness in various stages of CKD, the best combination strategies, and its mechanisms of action in humans. Biomarker-based strategies and precision medicine have the potential to further optimize future applications. Overall, semaglutide targets metabolic, inflammatory, and fibrotic pathways in DKD and could be a promising therapeutic agent to change patient outcomes and reduce the worldwide burden of this disease.

## Figures and Tables

**Table 1:** Mechanistic pathways of semaglutide-mediated renoprotection and supporting preclinical and clinical evidence.

Mechanism	Key Effects	Experimental Model/Evidence	Reference
Oxidative stress and inflammation	↓ ROS, ↑ SOD, ↓ TNF-α, IL-1β, IL-6	Obese mice with renal injury	[42]
Ferroptosis modulation	↑ β-Klotho, ↑ AMPK, modulates iron metabolism, fatty acid synthesis, lipid peroxidation; ↓ ferroptosis	DKD mouse model	[43]
Structural renal protection	↑ podocyte slit density, ↓ glomerulosclerosis, ↓ KIM-1	Mouse model of hypertension-accelerated DKD	[44]
EV-mediated inflammation	↓ macrophage activation, ↓ pro-inflammatory cytokines	Human vitreous samples from type 2 diabetic and non-diabetic donors	[45]
Systemic metabolic effects	↓ glucose, ↓ lipid toxicity, ↓ inflammation	Transgenic mouse models with endothelial and hematopoietic cell-specific GLP-1 receptor deletion	[46]
Renal hemodynamics	↑ natriuresis, ↑ diuresis via NHE3 inhibition	Male Wistar rats	[47]

Note: ↑ indicates an increase or improvement; ↓ indicates a decrease or reduction.

**Table 3:** Comparison of randomized controlled trials (RCTs) and real-world observational studies (RWE) evaluating semaglutide in T2DM and DKD.

Feature	Randomized Controlled Trials (RCTs)	Real-World Observational Studies (RWE)
Study Design	Prospective, randomized, controlled (e.g., SUSTAIN-6, PIONEER-5, PIONEER-6)	Observational, non-randomized, pragmatic (e.g., Spanish multicenter cohort, CKD real-life study)
Population	Narrow inclusion criteria; selected T2DM patients with CVD or CKD	Broad, heterogeneous T2DM and CKD populations, including multiple comorbidities
Sample Size	Typically large for efficacy endpoints (e.g., >3000 participants in SUSTAIN-6)	Often smaller cohorts (e.g., 122–156 patients in Luna et al. [53]), but can vary widely
Follow-up	Usually limited to 1–2 years	Can be extended depending on clinical practice (up to 24 months in real-world studies)
Endpoints	Hard clinical endpoints: ESKD, eGFR decline, macroalbuminuria	Less standardized endpoints
Limitations	May not reflect the general population; strict inclusion criteria	Confounding factors, lack of randomization, and smaller sample sizes
Strengths	High internal validity; robust causal inference	Reflects clinical practice, real-world effectiveness, and external validity

**Table 4:** Comparative summary of semaglutide, SGLT2 inhibitors, and MRAs in DKD.

Drug Class/Agent	Mechanisms of Action	Renal Outcomes	Safety Concerns
Semaglutide (GLP-1RA)	Anti-inflammatory, anti-fibrotic, and natriuretic effects; weight loss, appetite regulation; improved glycemic control; reduction in oxidative stress	Decreases albuminuria; potential slowing of DKD progression; direct eGFR protection less established	Low risk of electrolyte imbalance; gastrointestinal side effects; rare risk pancreatitis
SGLT2 inhibitors (canagliflozin, dapagliflozin, empagliflozin)	Inhibits renal glucose reabsorption leading to glycosuria; reduces glomerular hyperfiltration via afferent arteriolar vasoconstriction; lowers intraglomerular pressure and systemic BP; reduces renal hypoxia	Robust reduction in risk of ≥50% eGFR decline, ESKD, renal or cardiovascular death (e.g., CREDENCE, DAPA-CKD, EMPA-KIDNEY)	Genital infections; risk of volume depletion; rare diabetic ketoacidosis; modest effects on weight and glycemia
Non-steroidal MRAs (finerenone)	Mineralocorticoid receptor antagonism → antifibrotic and anti-inflammatory effects; reduces renal and cardiovascular damage	Decreased risk of CKD progression and cardiovascular events; ~18% reduction in ESKD or persistent eGFR decline	Hyperkalemia requires monitoring; less pronounced metabolic effects

**Table 5:** Knowledge gaps and research priorities for semaglutide in DKD.

Gap	Research Priority	Expected Impact
Long-term renal outcomes	FLOW trial and its published results assessing eGFR slope, ESKD, and renal mortality	Establish definitive renoprotective efficacy
Advanced CKD/ESRD	Dose-finding, tolerability, and safety studies in eGFR < 30 mL/min/1.73 m^2^ and dialysis populations	Guide safe use in high-risk patients
Biomarker-guided response	Validation of urinary (KIM-1, NGAL) and plasma (TGF-β) biomarkers; multi-omics and imaging studies	Enable precision medicine and monitor treatment response
Mechanistic understanding	Clinical studies on anti-inflammatory, antifibrotic, and hemodynamic effects; renal biopsy and tissue-level imaging	Clarify mechanisms and optimize therapy targeting
Combination therapy	RCTs with SGLT2 inhibitors + MRAs and/or GLP-1RA + SGLT2i to assess additive or synergistic benefits	Inform clinical decision-making for dual/triple therapy
Real-world evidence	Observational studies in diverse, comorbid populations	Ensure generalizability and safety across populations

## Data Availability

No new data were created or analyzed in this study.
